# Molecular cytogenetic (FISH) and genome analysis of diploid wheatgrasses and their phylogenetic relationship

**DOI:** 10.1371/journal.pone.0173623

**Published:** 2017-03-09

**Authors:** Gabriella Linc, Eszter Gaál, István Molnár, Diana Icsó, Ekaterina Badaeva, Márta Molnár-Láng

**Affiliations:** 1 Department of Plant Genetic Resources, Agricultural Institute, Centre for Agricultural Research, Hungarian Academy of Sciences, Martonvásár, Hungary; 2 Vavilov Institute of General Genetics, Russian Academy of Sciences, Moscow, Russia; Leibniz-Institute of Plant Genetics and Crop Plant Research (IPK), GERMANY

## Abstract

This paper reports detailed FISH-based karyotypes for three diploid wheatgrass species *Agropyron cristatum* (L.) Beauv., *Thinopyrum bessarabicum* (Savul.&Rayss) A. Löve, *Pseudoroegneria spicata* (Pursh) A. Löve, the supposed ancestors of hexaploid *Thinopyrum intermedium* (Host) Barkworth & D.R.Dewey, compiled using DNA repeats and comparative genome analysis based on COS markers. Fluorescence *in situ* hybridization (FISH) with repetitive DNA probes proved suitable for the identification of individual chromosomes in the diploid JJ, StSt and PP genomes. Of the seven microsatellite markers tested only the (GAA)_n_ trinucleotide sequence was appropriate for use as a single chromosome marker for the *P*. *spicata* A^S^ chromosome. Based on COS marker analysis, the phylogenetic relationship between diploid wheatgrasses and the hexaploid bread wheat genomes was established. These findings confirmed that the J and E genomes are in neighbouring clusters.

## Introduction

The tribe *Triticeae* contains nearly 100 annual species including agronomically important domesticated crops such as wheat, which is globally the third most-produced cereal after maize and rice. Approximately 400 perennial grasses with diverse genetic architecture also belong to the *Triticeae*. Five major genomic types (St, P, Ns, J and E) can be discriminated in the diploid and polyploid species. The genus *Agropyron* (crested wheatgrass) includes 10–15 species, of which the diploids, tetraploids and hexaploids are all based on the P genome. The genus *Pseudoroegneria* (bluebunch wheatgrass) was built around the common genome St, which is one of the most important basic genomes of perennial *Triticeae* species. Within the genus *Thinopyrum* (tall and intermediate wheatgrass), about 20 diploid, allotetraploid, allohexaploid, octoploid and decaploid species possess the J, E and sometimes St genomes [1].

As a tertiary gene pool for hexaploid wheat, perennial diploid wheatgrass species are important genetic resources and attracted researchers’ attention many decades ago because of their favourable and mostly untapped genetic background. Perennial grasses are highly tolerant against abiotic stress, such as low temperature and drought [2,3], salinity and waterlogged conditions [4,5] and are resistant to several diseases, such as leaf and stem rusts, Wheat Streak Mosaic Virus, Barley Yellow Dwarf Virus, Fusarium Head Blight, etc. [6–8]. These agronomic traits can be transferred into cultivated wheat, because its chromosomes pair well with chromosomes from the *Agropyron*, *Pseudoroegneria*, *Psathyrostachys*, *Thinopyrum*, *Elymus* and *Leymus* genera at meiosis [9]. As a consequence, many resistance genes (*Lr19*, *Lr29*, *Sr24/Lr24*, *Sr26*, *Lr38*, etc.) were identified in wheatgrass species and successfully transferred into wheat by interspecific hybridization [10–12]. On the other hand, apart from extensive research efforts aimed at the chromosome-mediated transfer of wild alleles, the genetic potential of perennial grasses is still largely underutilized in wheat breeding programs.

Limitations to the use of wild alleles in wheat breeding are related to the time- consuming development of alien introgression lines. To date, relatively low-throughput cytogenetic methods, such as C-banding [10], and especially fluorescence in situ hybridization (FISH) [13–15] and genomic in situ hybridization (GISH) [16,17], have been the most popular techniques for the detection and characterization of alien introgressions. FISH using repetitive DNA probes has made it possible to identify individual chromosomes within a species [18,19] and reveal intergenomic chromosome rearrangements [20]. Detailed FISH-based karyotypes have been developed for *Aegilops* species using DNA repeats [21,22]. To date, a detailed FISH-based, diploid wheatgrass karyotype is only available for the E genome [23], greatly limiting the efficiency of FISH to identify the chromosomes of perennial grasses and their segments. The cytomolecular characterization of diploid taxa could make it possible to use new FISH probes, to understand the karyotypic evolution of all the polyploid species mentioned above [23,24] and to investigate phylogenetic relationships between the perennial *Triticeae* genomes and wheat.

Genetic changes happening during the evolution of different *Triticeae* species may disrupt the collinearity between homoeologous wheat and alien chromosomes, as observed in taxa of *Aegilops* and *Secale* [25–27]. It could mean that compensating the loss of wheat genes did not happened, which could influence the agricultural efficiency of wheat-alien introgressions in the future. The contradictory opinion on phylogenetic relationships between the genomes of wheat and perennial grasses also hampers the utilization of wild genetic diversity in wheat breeding.

Cytogenetic studies, mainly based on the analysis of meiotic chromosome pairing in diploid and triploid hybrids indicated that *Thinopyrum bessarabicum* (Savul.&Rayss) A. Löve and *Thinopyrum elongatum* (Host) D.R.Dewey should be placed in separate genera [28,29], while other studies indicated that the genomes of *T*. *bessarabicum* and *T*. *elongatum* are similar enough to be included in a single genus [30–33].

By the use of sequence-based markers including single- (or low-) copy nuclear genes, such as the β-amylase gene, Mason-Gamer [34] placed *T*. *elongatum* in a well-supported clade within *Aegilops* and *Triticum*, while *T*. *bessarabicum* could be found at the base of this clade. In the same study, *Agropyron cristatum* (L.) Beauv. and *Pseudoroegneria spicata* (Pursh) A. Löve were included in two separate clades [34]. The use of Granule-bound starch synthase I (GBSSI) sequences, another nuclear gene, showed that E-, J- and P-genome species are more closely related with *Triticum*/*Aegilops* taxa than the St-genome *Pseudoroegneria* species [35]. Recently, Wang and Lu [36] summarized the available results of phylogenetic studies made on perennial and annual *Triticeae* species. Different DNA sequence-based assays indicated that both the J and E genomes, only the genome J, or only the E genome were close to the A/B/D genomes of the *Triticum*/*Aegilops* complex, but in some cases neither the J nor the E genome was closely related to the A/B/D genomes. It was also reported that the St genome of *Pseudoroegneria* and the P genome of *Agropyron* were moderately related to the J/E genome [36]. Based on these results it is still not clear whether the J (Eb) and E (Ee) genomes are in the same, neighbouring or distantly separated clusters, and how these genomes are related to the St and P genomes and to the A/B/D genomes of wheat.

One disadvantage of applying sequence-based markers for a single gene family is that only the variability within a small part of the genome is considered when comparing different species [37]. Genes conserved throughout evolution in terms of both sequence and copy number make it possible for large complex genomes to be compared at multiple loci. Conserved orthologous set (COS) markers were identified by the *in silico* comparison of rice, wheat and *Brachypodium* ESTs [38]. As they target to the exon-intron boundaries of genes conserved between the model and target species, COS markers are highly polymorphic. Orthologous regions on the chromosomes, represented by these markers, enable the genomes of rice, wheat, maize, sorghum, barley and *Aegilops* to be compared [27,39,40].

A set of COS markers covering the homoeologous group 1–7 chromosomes of wheat, combined with in situ hybridization using various repetitive probes provide a powerful set of tools for clarifying complex genomic relationships such as that between wheat and perennial grasses.

Detailed FISH-based karyotypes of each diploid wheatgrass species would make the rapid cytogenetic analysis of genetic materials possible. It is very difficult to follow gene transfer in different genetic lines carrying wheatgrass chromosomes or chromosomal segments in a wheat background because of the lack of molecular and cytogenetic markers. The aim of the present study was to set up detailed FISH karyotypes for three diploid wheatgrass species and to determine individual chromosomal markers. By comparing FISH and molecular marker data, a closer phylogenetic relationship between these genomes was highlighted.

## Materials and methods

### Plant material

**Fig 1:**
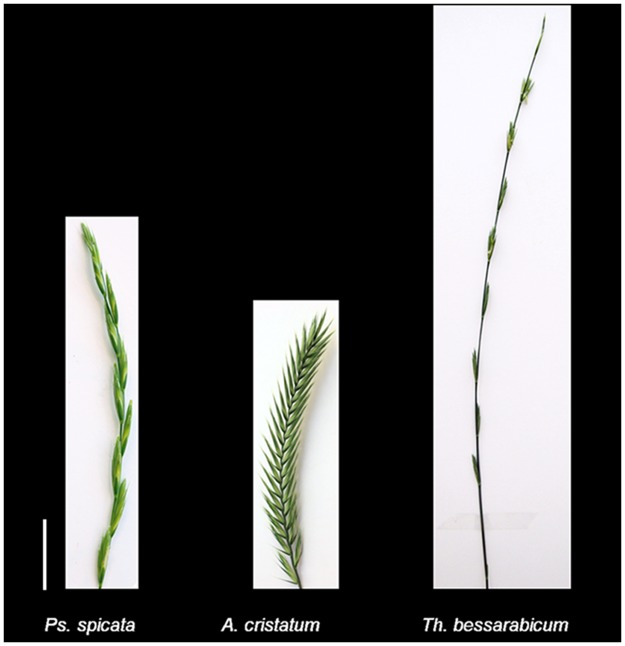
Spike morphology of three representative diploid wheatgrass plants. All plants were grown under greenhouse conditions in Martonvásár, Hungary (Size marker = 10mm).

Nine accessions belonging to four diploid species, *Thinopyrum elongatum* D.R. Dewey (2n = 2x = 14, EE), *Agropyron cristatum* (L.) Beauv. (2n = 2x = 14, PP), *Thinopyrum bessarabicum* (Savul. & Rayss) A. Löve (2n = 2x = 14, JJ or EbEb) and *Pseudoeregneria spicata* (Pursh) A. Löve (2n = 2x = 14, SS or StSt) (Fig 1) were included in the present study together with the wheat (*Triticum aestivum* L.) cultivar ‘GK Öthalom’ (Table 1). Plant material seeds and individual plants from each genotype originally obtained from the Genebank, USDA-ARS Beltsville, Md., USA and the genetic collection of the Moscow Scientific-Research Agricultural Institute “Nemchinovka”, Moscow, Russia, are now maintained at the Cereal Genebank and Perennial Garden, Department of Plant Genetic Resources, Martonvásár, Hungary.

**Table 1:** Original (in brackets) and Martonvásár (MvGB) registration number and country (area) of geographical origin for accessions used for FISH and COS marker analysis. Accession number 362480 is maintained in Moscow Scientific-Research Agricultural Institute “Nemchinovka”, Russia.

Species	Accession no.	Country of origin
***Agropyron cristatum***	(PI 639814)MvGB 1521	Mongolia
	(PI 636511)MvGB 1509	Bulgaria
***Pseudoroegneria spicata***	(PI 610973)MvGB 1607	Unknown
	(Unknown)362480	Unknown
	(PI 618736)MvGB 1605	Unknown
***Thinopyrum bessarabicum***	(PI 531711)MvGB 1705	Estonia
	(W6 10232)MvGB 1703	Russia
	(PI 531712)MvGB 1706	Ukraine
***Thinopyrum elongatum***	(PI571718)MvGB 1963	Tunisia

### McFISH analysis

Seed germination and root tip mitotic chromosome preparations were carried out according to Linc et al. [20].

Multicolor fluorescence *in situ* hybridization (mcFISH) was performed using standard repetitive DNA sequences, a telomere specific repeat (HT100.3) and seven microsatellite motifs, as listed in Table 2.

**Table 2:** DNA repetitive probes and trinucleotide sequences used for FISH karyotyping.

Species	*A*. *cristatum*		*T*. *bessarabicum*			*P*. *spicata*	
Accession no.	MvGB1521	MvGB1509	MvGB1705	MvGB1703	MvGB1706	MvGB1607	MvGB1605
**pSc119.2** Bedbrook et al. (1980)	+	+	+	+	+	+	+
**Afa-family** Nagaki et al. (1995)	+	+	+	+	+	+	+
**pTa71** Gerlach and Bedbrook (1979)	+	+	+	+	+	+	+
**pAs1** Rayburn and Gill (1987)	+	+	+	+	+	+	+
**HT100.3** Juchimiuk-Kwasniewska et al. (2011)	+	+	+	+	+	+	+
**(GAA)**_**n**_ Vrana et al. (2000)	-	-	-	-	-	+	+
**(CAC)**_**n**_ Vrana et al. (2000)	-	-	-	-	-	-	-
**(AGG)**_**n**_ Vrana et al. (2000)	-	-	-	-	-	-	-
**(ACT)**_**n**_ Vrana et al. (2000)	-	-	-	-	-	-	-
**(ACG)**_**n**_ Vrana et al. (2000)	-	-	-	-	-	-	-
**(AAC)**_**n**_ Vrana et al. (2000)	-	-	-	-	-	-	-
**(CAG)**_**n**_ Vrana et al. (2000)	-	-	-	-	-	-	-

The probe pTa71(45S rDNA) contains a 9.05 kbp fragment, which is part of an rDNA repetitive unit consisting of one copy each of 18S rDNA, 5.8S rDNA and 25S rDNA, and an intergenic spacer from wheat cv. Chinese Spring [41]. The probe pAs1 contains a 1-kb DNA fragment isolated from *Aegilops tauschii* Coss. in the plasmid pUC8 [19]. The Afa-family repeats [42] were amplified from the genomic DNA of barley (*Hordeum vulgare* L). The DNA repetitive clone pSc119.2, inserted into the plasmid pBR322, contains a 120 bp repeat derived from an *Eco*RI relic DNA from rye cv. King II [43]. The HT100.3 telomere repeat (TTTAGGG)n sequences were originally isolated and amplified from *Arabidopsis thaliana* L. [44]. The Fat probe was labelled with Fluorescein by PCR amplification from the 3B_050_N05 BAC clone using primers GGGGAGCTTCTCACAACAAGC and TATTTACCACGGCATGTCGGG resulting in an approximately 460-bp fragment [45]. Microsatellite probes were amplified from wheat genomic DNA according to Vrana et al [46].

For 3-color FISH, the pSc119.2 and Afa-family DNA sequences were amplified and labelled by PCR either with biotin-11-dUTP (Roche) or with biotin-14-dATP (Invitrogen) and digoxigenin-11-dUTP (Roche) by means of Nick Translation. The clone pTa71 was labelled combinatorially with 50% biotin-11-dUTP and 50% digoxigenin-16-dUTP. All seven microsatellite repeat sequences were amplified from genomic DNA of *T*. *aestivum* and labelled by PCR with biotin-11-dUTP and digoxigenin-11-dUTP. Digoxigenin and biotin were detected using anti-digoxigenin-Rhodamine Fab fragments (Roche) and Alexa Fluor-488 Streptavidin (Invitrogen).

*In situ* hybridization was carried out on mitotic chromosome spreads of each *Agropyron* species according to Linc et al. [23], with minor modifications. The hybridization solution per slide (20 μl) contained 50% formamide, 2×SSC, 10% dextran sulfate, 0.1% sodium dodecyl sulfate, 50 ng/μl carrier DNA, and a combination of two or three ~300 pg/μl fluorochrome-labeled probes. The slides were treated for 2 min at 80°C followed by hybridization overnight at 37°C. After documenting the hybridization patterns of the microsatellite probes, the slides were rinsed off (3×6 min in 50% formamide and 2×SSC at 42°C) and re-hybridized with a combination of two different repetitive DNA sequences under the same conditions as described above. The chromosomes were counterstained with 2μg/ml DAPI (4’-6-diamino-2-phenylindole) and mounted in antifade solution (Vectashield, Vector Laboratories).

Fluorescent signals were visualized using a Zeiss AxioImager M2 epifluorescence microscope equipped with filter sets for detecting DAPI, FITC and Rhodamine signals. Images were captured with a Zeiss AxioCam MRm CCD camera and processed with Zeiss AxioVision 4.8.2. software. All the images presented in this paper were manipulated only to enhance contrast.

The chromosomes of diploid wheatgrass species were classified according to similarity of their FISH patterns and designated A-G with a genomic symbol given as superscript.

### COS marker analysis

Genomic DNA was extracted from fresh young leaves of hexaploid wheat (Hungarian variety GK Öthalom), *T*. *elongatum* (Host) Nevski (MvGB1965), *T*. *bessarabicum* (MvGB1706), *P*. *spicata* (MvGB1607, MvGB1615) and *A*. *cristatum* (MvGB1521, MvGB1509) using a QuickGene DNA Tissue Kit (FujiFilm, Japan) according to the manufacturer’s instructions. The DNA concentrations were adjusted to 50 ng μl^-1^.

A total of 117 markers, specific for wheat homoeologous groups 1–7, representing both chromosome arms of each analyzed accessions were chosen from a publicly available COS marker collection published by Quraishi et al. [38] and summarised in S1 Table. PCR reactions with primers for COS markers were performed in a 12 μL reaction volume as described by Molnár et al. [47] using the touchdown reaction profile: 94°C (2 min), 10 cycles of (94°C (0.5 min), Ta +5°C (0.5 min) decreasing in 0.5°C increments for every subsequent set of cycles, 72°C (1 min), 30 cycles of (94°C, 0.5 min), Ta°C (0.5 min), 72°C (1 min), hold at 72°C (2 min).

PCR amplicons have been separated by a Fragment Analyzer Automated CE System equipped with a 96-Capillary Array Cartridge (effective length 33 cm) (Advanced Analytical Technologies, Ames, USA) and analyzed using PROsize v2.0 software. The annealing temperature (Ta) for each COS marker, together with data for the PCR amplicons, are also included in S1 Table.

### Phylogenetic analysis

The banding patterns of the PCR products were scored as present (1) or absent (0) for each marker. A dendrogram was constructed using the Cluster 3.0 program and visualized with Java TreeView 1.1.6r4. Genetic similarities between the 7 accessions/genotypes were measured using Jaccard’s similarity coefficient based on the proportion of shared alleles. The Jaccard’s similarity coefficients were calculated as follows: J(i_1_,i_2_) = a/(a+b+c) [48], where *a* is the number of bands shared by both individual samples (i_1,_ i_2_), *b* is the number of bands where i_1_ has a band, but i_2_ does not, *c* is the number of bands where i_2_ has a band, but i_1_ does not.

## Results

### FISH-based karyotype analysis

In the present study three diploid wheatgrass species were used for FISH-based karyotype analysis. Two populations (accessions) of the species *A*. *cristatum* and 3 populations each of *P*. *spicata* and *T*. *bessarabicum* with different geographical origin were examined (Table 1).

**Fig 2:**
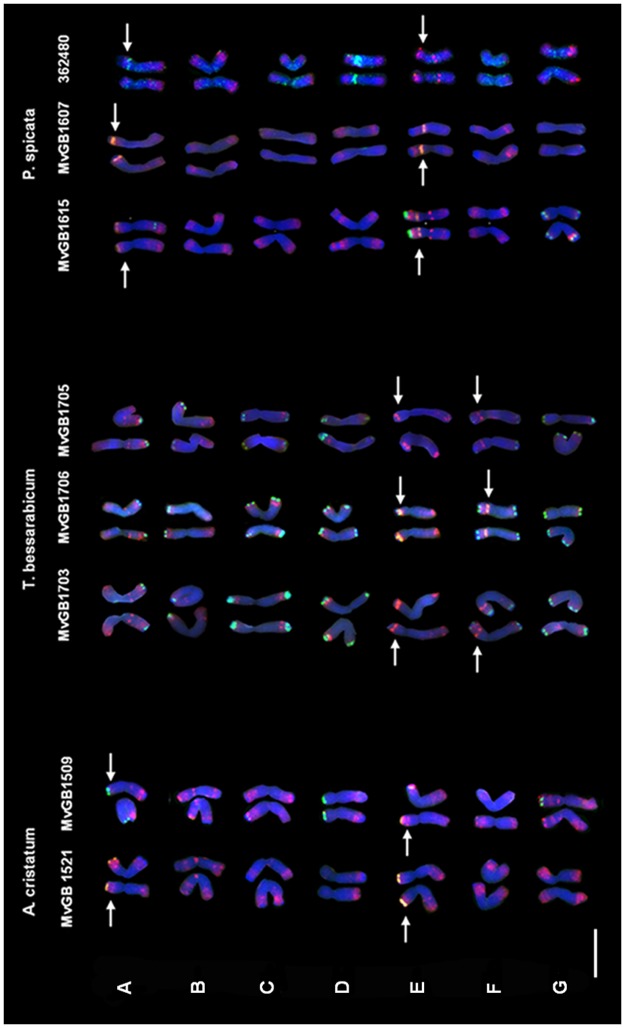
Chromosome FISH- karyotype of three diploid wheatgrass species. Repetitive DNA sequences were used as markers to construct karyotypes: pSc119.2 green, Afa- family red and pTa71 (45S rDNA) orange (pAs1 and HT100.3 not shown) *P*. *spicata* accession 362480 chromosomes show repetitive DNA markers pAs1 red and Fat probe green. Arrows indicate positions of NOR loci. Scale bar = 10μm.

**Fig 3:**
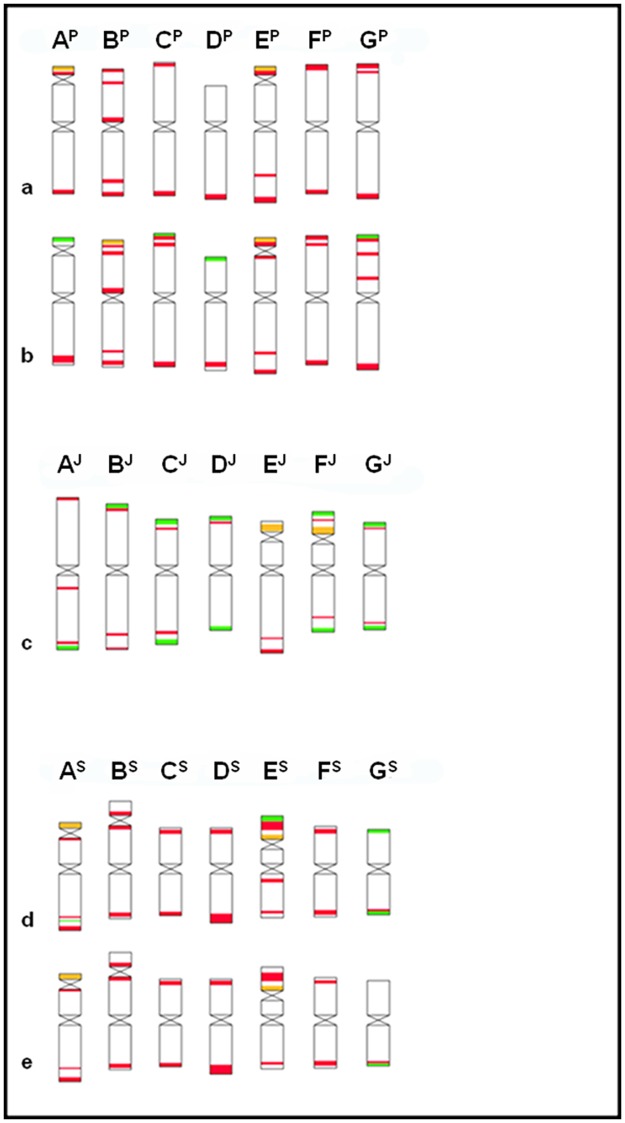
FISH-banded idiograms of P-, J- and St- genome chromosomes. The most representative FISH bands from each accession were used to construct the idiograms (pSc119.2 green, Afa- family red and pTa71 (45SrDNA) orange). *A*. *cristatum*, (a) MvGB1521, (b) MvGB1509; *T*. *bessarabicum* (c) MvGB1703, 1705, 1706; *P*. *spicata* (d) MvGB1615, (e) MvGB1607.

Detailed FISH karyotypes of the diploid species chromosomes were generated using repetitive DNA sequences after validating the chromosome morphology and arm ratios of at least 30 metaphase cells from each accession (Fig 2). Chromosome idiograms were also constructed after detailed studies of chromosome morphology (Fig 3). The simultaneous hybridization of the repetitive DNA probes Afa family (pAs1 for accession 362480); pSc119.2 (Fat probe for accession 362480) and pTa71 on mitotic metaphase cells made it possible to characterize single chromosomes of the three diploid wheatgrass species. Certain variability in the number and position of FISH signals was observed for some homologous chromosomes in different plants of each of the diploid genotypes. However, these polymorphisms did not hamper the precise characterization of all the individual chromosomes; the labelling patterns were species- and chromosome- specific.

### Agropyron cristatum

All seven P genome chromosomes of both accessions carried specific pSc119.2 and Afa signals, so they could all be distinguished from each other. However, this species had the least characteristic chromosomes based on their FISH patterns, and many chromosome polymorphisms affecting all seven chromosome pairs were detected between the two accessions. Two of the seven chromosome pairs possessed NOR regions. The E^P^ chromosome had a clearly distinguishable satellite in both accessions, while the satellite on A^P^ was much narrower and was only visible in MvGB 1521 genotype. The Afa signals on E^P^ appeared mainly in terminal and subterminal positions in both accessions. Instead of showing a pTa71 signal on A^P^S, MvGB1509 had a characteristic pSc119.2 site at the chromosome tip. Chromosome B^P^ was found to be (sub)metacentric and carried Afa-family sites on both arm, mainly terminally (B^P^S) and subterminally (B^P^S). There was a characteristic Afa signal on B^P^S in the pericentromere region. C^P^, a large metacentric chromosome, had mainly Afa-family signal on both arms in subterminal and terminal positions. There was a faint pSc119.2 signal at the tip of C^P^S, but only in the MvGB1521accession. The submetacentric chromosome D^P^ carried Afa signals in both accessions, in subterminal-terminal positions. A strong pSc119.2 signal was located at the tip of D^P^S in accession MvGB1521. The submetacentric chromosome F^P^ possessed Afa-family repeats on both arms, in terminal-subterminal positions. The G^P^ submetacentric chromosome showed strong FISH-pattern polymorphism between the two chromosomes of the accessions analyzed. In MvGB1509, G^P^S carried a faint Afa-family signal at the pericentromere and a pSc119.2 signal at the tip of the chromosome arm.

### Thinopyrum bessarabicum

All the chromosomes could be distinguished according to their FISH patterns. The number and position of signals was similar for all three accessions, so this species had the least chromosomal polymorphism among the three diploid genomes investigated. In contrast to the other genome types, the J-genome chromosomes carried either one or, more frequently, two distinct pSc119.2 signals in the telomeric regions of their short and/or long arms (Fig 2). The A^J^ metacentric chromosome possessed a clear terminal pSc119.2 site on the long arm and diffused subterminal Afa signals on both arms. A distinct intercalary Afa signal in the proximal third of the long arm was characteristic of chromosome A^J^, which could be detected in all the accessions analyzed. The B^J^ submetacentric chromosome showed a strong pSc119.2 signal on the short arm and faint, diffused subtelomeric Afa-family signals on both arms. Large terminal pSc119.2 signals were visible on both arms of the C^J^ (sub)metacentric chromosome, and a strong subterminal Afa cluster was detected on the long arm. Terminal pSc119.2 signals were detected on both arms of the metacentric D^J^ chromosome, which also carried a distinct subterminal Afa cluster on the short arm. Chromosome E^J^ had a small satellite, which only showed a terminal pSc119.2 site in one accession. A large Afa cluster was observed in the perinucleolar region on the short arm and fuzzy signals were detected in a subterminal position on the long arm, with intensities varying among the accessions. Very weak, diffused signals of the Afa repeat were dispersed over the distal half of the E^J^ long arm. The F^J^ chromosome was characterized by its large satellite. Both the satellite and the long arm were terminated by strong pSc119.2 signals. A large, bright Afa cluster was located in a perinucleolar position and much weaker, diffused signals of this probe were found in the distal parts of both arms. Both satellite chromosomes, E^J^ and F^J^, carried a 45S rDNA locus on their short arms (more fainted signals on MvGB1705 chromosomes). The G^J^submetacentric chromosome showed strong telomere pSc119.2 signals on both arms, and relatively faint, diffused Afa signals in the subterminal regions. The signals on the long arm were a little more intense than those on the short arm.

### Pseudoroegneria spicata

The A^S^ chromosome was submetacentric with a narrow satellite on the short arm. Strong, fuzzy Afa signals were found in the subterminal region of the long arm, extending toward the centromere, up to the middle part of the arm, though the intensity of these was much weaker. The Afa-family signal on the short arm was weaker and the pSc119.2 probe was totally absent from the satellite arm. Interestingly enough, only one of the two homologous A^S^ chromosomes carried specific pSc119.2 signal in a subterminal position on the long arm. The B^S^ chromosome had a secondary constriction, which failed to show a pTa71 signal, but possessed a strong DAPI-positive band. Large DAPI bands were also found at both termini of the chromosome. Bright, diffused *Afa*-family signals were observed on both arms, adjacent to the DAPI bands. A distinct DAPI band was present terminally on the short arm of the C^S^ chromosome; diffused Afa-family signals were observed in the subterminal position on the short arm, adjacent to the DAPI band, and in the terminal position on the long arm. D^S^ is a submetacentric chromosome with two DAPI-positive bands, a larger one on the short arm and a smaller one on the long arm). Afa-family signals were found subterminally on both arms but the signal intensity was greater on the long arm. E^S^ had a secondary constriction in the proximal third of the short arm, which coincided with the location of the pTa71 signal. This chromosome showed the most complex FISH pattern in all the accessions investigated. Both arms carried clear terminal DAPI-positive bands. A strong pSc119.2 signal was found terminally on the satellite and a strong, fuzzy Afa-family signal on the distal half. This labelling pattern was detected on the E^S^ chromosome of the accessions MvGB1615 and PI 362480, but the large proximal Afa cluster was absent from one of the homologous chromosomes of the MvGB1607 accession. A clear-cut Afa cluster was present in the very proximal part of the long arm arm rather faint, fuzzy signals of this probe were found in a subterminal position, adjacent to the DAPI band. Two distinct DAPI bands were also detected in a subterminal position on the short and long arms of the F^S^ and G^S^ chromosomes. Bright, diffused Afa-family signals with approximately the same intensity were detected on both arms of F^S^, close to the DAPI bands. The G^S^ chromosome showed a strong pSc119.2 signals on both arms (only on G^S^L of MvGB1607) but the intensity of both subterminal Afa-family signal was very low.

In addition to the Afa- family, the line PI 362480 was also subjected to FISH analysis with the Fat probe. Its Afa- karyotype was in good agreement with the chromosome patterns of the two other accessions. One chromosome pair was characterized by an abundance of Fat signals in the pericentromeric region, which is typical of group 4 chromosomes [45]. Based on the Afa pattern and the position of the large DAPI band, this chromosome corresponded to B^S^ (current classification).

### Microsatellite repeat analysis

Two-step fluorescence in situ hybridization was used to identify the hybridization patterns of seven SSR motifs on the chromosomes of three wheatgrass species. The first hybridization was carried out with the microsatellite probes (GAA)n, (ACT)n, (CAG)n, (AGG)n, (CAC)n, (AAC)n and (ACG)n. After the documentation of the FISH sites (if necessary), the slides were re-hybridized with the standard repetitive DNA probes pSc119.2, pTa71 and Afa- family in order to characterize individual chromosomes with the specific microsatellite location. Among the seven microsatellite repeats studied, only (GAA)n produced a distinct FISH signal on the A^S^ short arm of the two accessions *P*. *spicata* (MvGB1607 and MvGB1605, not showed). Based on these results, the (GAA)n SSR motif can be used as an individual A^S^ chromosome- specific cytogenetic marker. None of the other microsatellite repeats were suitable for use as molecular cytogenetic markers for the characterization of the J, St and P genomes.

### COS marker analysis

COS markers specific for wheat homoeologous groups 1–7 were used for PCR analysis of the total genomic DNA of the perennial species in order to provide tools for detecting the genomes E, J, St and P in the wheat genetic background and to establish their genetic similarity relative to wheat. Among the 117 COS markers studied, 102 showed PCR products in the wheat control genotype (GK Öthalom) or in at least one of the 6 wheatgrass lines, while 15 markers amplified no product. The 102 markers (wheat chromosome group 1: 12, group 2: 11, group 3: 18, group 4: 10, group 5: 12, group 6: 19, group 7: 20) resulted in 396 bands of various sizes (S1 Table).

**Fig 4:**
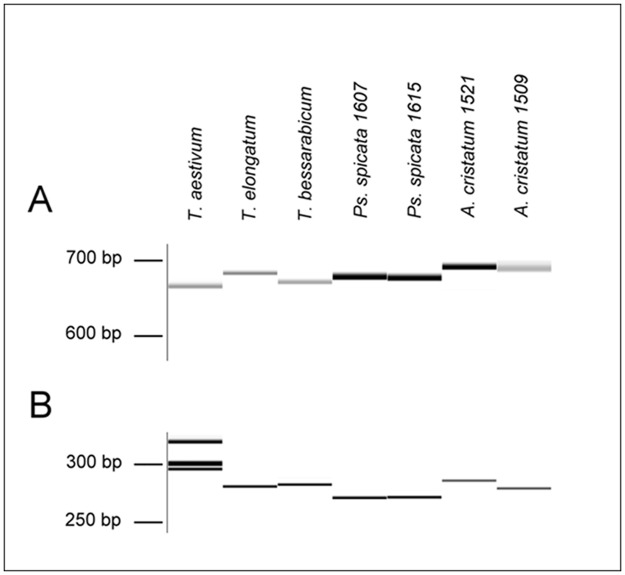
Examples of COS markers resulting in polymorphic PCR products between hexaploid wheat and perennial grasses. (A) Amplicons produced by the markers *c746642* and (B) *c756279* from genomic DNA of wheat genotype GK Öthalom (*T*. *aestivum*), *T*. *elongatum* MvGB1963, *T*. *bessarabicum* MvGB1706, *Ps*. *spicata* MvGB1607 and MvGB1615, and *A*. *cristatum* MvGB1521 and MvGB1509.

Out of the 119 loci (85 markers) detected in the E- genome of diploid *T*. *elongatum* 54 (43 markers) showed significant length polymorphism (≥5bp) relative to the wheat genotype GK Öthalom (Fig 4). In the case of *T*. *bessarabicum*, where 113 loci (81 markers) were located on the J- genome, 49 loci (44 markers) were polymorphic.

In the case of *P*. *spicata* and *A*. *cristatum* two accessions of each species were investigated and only amplicons exhibiting similar size within the species but size polymorphism relative to wheat were considered to be suitable for pre-breeding purposes. In *P*. *spicata* genotypes MvGB1607 and MvGB1615, 123 and 140 loci, respectively, were assigned to the St genome, of which 62 loci (50 markers) showed significant size polymorphism. Finally, 42 loci (36 markers) were polymorphic among the 126 and 134 located on *A*. *cristatum* genotypes MvGB1521 and MvGB1509 (P-genome), respectively.

Genome-specific loci of the COS markers with significant (≥5bp) length polymorphism between wheat genotype GK Öthalom and the perennial grasses were considered to be suitable for the marker-assisted selection of new wheat-alien introgression lines in pre-breeding programs. In this study, 207 polymorphic loci of 76 markers were found to be potentially useful for the detection of the E, J, St or P genomes of perennial grasses.

### Genetic similarity between perennial grasses and wheat

**Fig 5:**
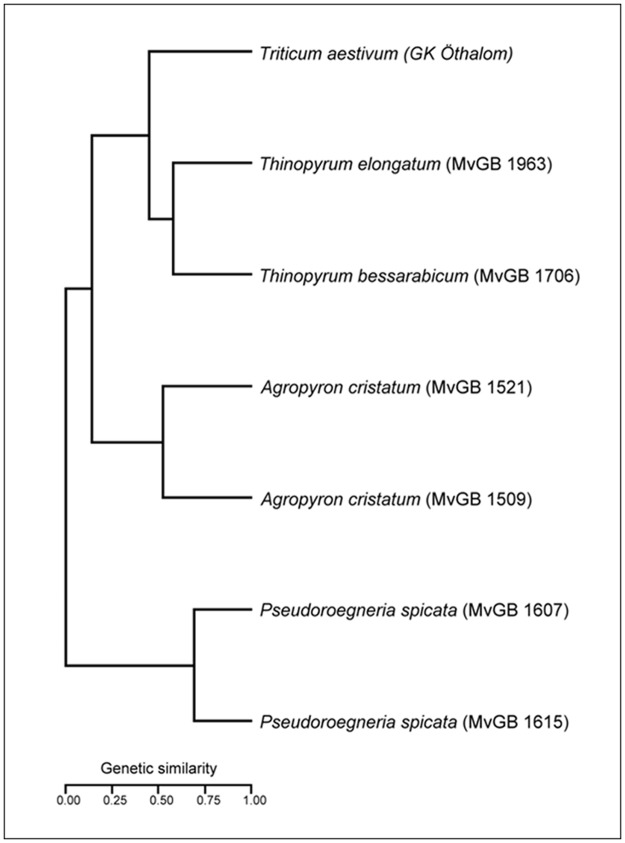
Dendrogram illustrating genetic similarities between the genera *Thinopyrum*, *Agropyron*, *Pseudoroegneria* and hexaploid wheat. Dendrogram was generated by UPGMA cluster analysis (Cluster) and calculated from 206 loci produced by 97 COS markers.

For phylogenetic analysis, the PCR amplicons were scored as present (1) or absent (0) for each marker and were used as character states. Amplicons of identical size were considered to be the same. Only bands shared between at least two genotypes were considered in the calculation of Jaccard’s similarity coefficient. A total of 206 loci of 97 COS markers were scored in a global matrix of which 188 (91%) were polymorphic between the species. The genetic similarities ranged from 0.26 to 0.74. Differences in the degree of similarity between species were confirmed by statistical analysis. The dendrogram generated from the analysis of similarity (Fig 5) separated most of the populations and all the species, which split into three groups. The first group included the E-genome species *T*. *elongatum*, the J-genome species *T*. *bessarabicum* and *T*. *aestivum*. Both genotypes of the P-genome species *A*. *cristatum* fell in the second group, while the two genotypes of the St-genome species *P*. *spicata* formed a third, distantly separated clade.

## Discussion

Perennial *Triticeae* species are important as tertiary gene pools for wheat breeding programs. However, detailed knowledge of the genetic and cytogenetic structure of these species is required for the targeted exploitation of their variability. The chromosomal positions of 45S and 5S rDNAs site have been determined for many perennial *Triticeae* species [1,49,50] and a detailed mcFISH karyotype has been constructed for the diploid E genome [23], but to date, no such FISH polymorphism experiments have been performed on other diploid *Thinopyrum* genomes. Far fewer species-specific cytogenetic and molecular markers are available than are needed for controlled, successful gene transfer between wheat and species belonging to the tertiary gene-pool of wheat.

The relationships between diploid perennials have always been an important subject of debate among scientists (taxonomists, cytogeneticists, etc.). Hsiao et al. [24] studied the karyotypes of 22 diploid perennial *Triticeae* species representing the P, St, J (E), H, I, Ns W and R genomes. Based on these earlier investigations, two satellite chromosomes of the J and E genomes were assigned to homologous groups 4 and 5, while Dvorak et al. [49] suggested that SAT chromosomes in the *Triticeae* might belong to groups 1, 5 and 6. Indeed, by using RFLP analysis of non-transcribed spacer of rRNA genes they assigned two satellite chromosomes of *Elytrigia elongata* (= *Thinopyrum elongatum*) to homoeologous groups 5 and 6, which was later confirmed by mcFISH of a set of ditelosomic and addition lines [23]. *T*. *bessarabicum* and *T*. *elongatum* genetic similarity based on previous studies is partially proved [36], therefore the satellite chromosomes of this species should also be placed to groups 5 and 6.

Position of rDNA signals on *A*. *cristatum* chromosomes however was different from what we have seen in *T*. *bessarabicum*: the signals of pTa71 probe were located in terminal regions of chromosome pairs A^P^ and E^P^, the latter one been classified as 5P by Said et al. [51]. These authors also showed that the second satellite chromosome classified here as A^P^ did not belong to group 1 and its relationship with wheat homoeologous chromosomes remained unknown. This chromosome did not possess 5S rDNA locus and could belong to group 6, although terminal position of rDNA locus was not typical for this group. Alternatively, the satellite chromosome A^P^ of *A*. *cristatum* might has derived from group 6 chromosome as a result of translocation of transposition of rDNA locus.

The C-banding patterns of 10 diploid species were studied earlier by Endo and Gill [52], who drew the attention to the equivalence/similarity of the J and E genomes already 30 years ago. These two genomes cannot be distinguished on the basis of chloroplast sequence data, but chromosome pairing analysis in meiosis, karyotype differences, and data on 5S rDNA spacer and ITS sequences provide clear evidence that these two species belong to different genera [28,37]. The genetic similarity between the E and J genomes still represents one of the biggest controversies in perennial *Triticeae* studies. Thus, according to the review of Wang and Lu [36], approx. half of the publications based on different assays put the J and E genomes in the same cluster, while the other half in adjacent or even in distantly separated clusters.

In the present FISH karyotype analysis, the single chromosomal FISH pattern of *T*. *bessarabicum* showed some polymorphisms between the accessions, but in most cases they exhibited a consistent picture. The current FISH-based picture of the J genome is obviously quite different from that of the E genome presented in a previous paper [23]. The most striking difference was found for the E^J^ and F^J^ chromosomes. While the 5E chromosome of *T*. *elongatum* had a distinct NOR and 6E failed to exhibit the 45S rDNA site, both the satellited chromosomes of *T*. *bessarabicum* (E^J^ and F^J^, exhibited clearly distinguishable 45S rDNA FISH signals on their short arms. This partially corresponds to the rDNA distribution in the *Triticeae* species [53].

Small 45S rDNA signals (NORs) are located in the subtelomeric regions of chromosomes A^P^ and E^P^ of *A*. *cristatum* and A^S^ of *P*. *spicata*. The satellites on *A*. *cristatum* chromosomes are very small, so they can hardly be expected to possess distinct secondary constrictions without special pre-treatment, similarly to the chromosomes of diploid wheat species, which rarely express distinct satellites [54]. The present investigation may explain earlier findings, which failed to reveal satellite chromosomes in the *A*. *cristatum* karyotype [51,54–56]. Li and Zhang [57] concluded that rDNA sites were unequal on homologous chromosomes of the J, St and E genomes, which partially correspond with the present data for the rDNA site on the P genome, where heteromorphism was observed on the A^P^ chromosome.

The FISH analysis of *P*. *spicata* using repetitive DNA revealed highly specific labelling patterns for all the S genome chromosomes. The signal of the 45S rDNA probe on the A^S^ chromosome was quite small and located in the subtelomeric region of the short arm in all the accessions. Murphy et al. [58] studied three accessions of *P*. *spicata*, and only in one did the A^S^ chromosome contain a satellite. The other accessions only showed a visible secondary constriction on chromosome E^S^, in agreement with the present results.

The polymorphism of repetitive sequences due to ancient hybridization followed by the incomplete sorting of ancestral polymorphisms (in addition to mutations, chromosome recombination events, etc.) could be responsible for intraspecific variation in diploid species [35]. The present results showed similar intraspecific variation in all three diploid wheatgrass genomes studied. The greatest intraspecific FISH polymorphism was detected on the P genome chromosomes (A^P^, D^P^, G^P^) and the lowest on the J genome chromosomes (F^J^). When FISH polymorphisms between J- and E- genome chromosomes assigned to two neighbouring clusters were compared, the validation of the karyotypes of four *T*. *elongatum* (E) accessions with different geographical origin showed much more extensive variations in the probe hybridization patterns (2E, 3E, 4E, 5E, 6E, 7E) [23] than those detected on the J genome chromosomes in the present study.

With one exception, all the microsatellite repeats tested failed to show any hybridization signals on the St-, J- and P- genome chromosomes. Apart from a few unidentifiable FISH signals, no chromosome arm-specific, characteristic signals were detected. The lack of these trinucleotide sequences is not unusual for the *Triticeae* species. An earlier study on wheatgrass genomes suggested that reliable chromosome identification could not be achieved using the GAA sequences [59]. However, the distribution of GAA and ACG clusters can be used as additional cytogenetic markers to characterize tetra- and diploid *Aegilops* chromatin in the wheat genetic background [22]. Moreover, with the use of GAA and CAG motifs, the 6A^m^ chromosome of *T*. *monococcum* can be clearly discriminated from other A genome chromosomes [60,61]. However, it has been proved that the number of hybridization sites may vary within a species (between individual accessions). It is probable that GAA sequences could only be useful as a molecular cytogenetic marker for perennial *Triticeae* species if a sufficient number of accessions per individual genome were tested. It was proved earlier [36,62] that the J/E and St genomes of perennial *Triticeae* species are evolutionary close to the ABD genomes of common wheat, and similar conclusions were drawn in the present FISH-based experiments and molecular marker analysis. This genome-based similarity explains the relative ease with which gene can be transferred from these genomes to the hexaploid wheat background [10–12]. This relationship could serve as a strong scientific background for successful gene transfers from wild *Triticeae* relatives carrying the J/E and St genomes for wheat improvement.

The results obtained on diploid wheatgrass species agreed well with previous studies and confirmed the high transferability of COS markers between species in the *Triticeae* [27,39,40,63,64]. The fact that 31–50% of the amplicons obtained on perennial wheatgrass species belonging to the tertiary gene pool were polymorphic relative to those obtained in hexaploid wheat suggested that a significant part of the genetic diversity in these wild species is due to the variability of intron regions [65]. Interestingly, using the same set of COS markers a similar ratio of polymorphic loci (46–53%) was obtained in diploid *Aegilops* species with the U, M, S or C genomes, which are more closely related to wheat [64].

In the present study, UPGMA cluster analysis based on 206 loci of 97 conserved orthologous nuclear genes showed that the E genome of *T*. *elongatum* and the J genome of *T*. *bessarabicum* are more closely related to hexaploid wheat than the P genome of *A*. *cristatum*, while the St genome of *P*. *spicata* is the most distantly related genome. These results are consistent with those of Mahelka et al. [35], who found the same genetic relationships between these species based on one nuclear gene (GBSSI) sequence. More recently, Hu et al. [66] developed 258 EST and 46 PLUG (PCR-based Landmark Unique Gene) primer pairs and found 43 markers suitable for distinguishing *T*. *elongatum* chromatin from the wheat genomes. The comparison of molecular marker locations on the chromosomes indicated that the E genome is closely related to the D genome of wheat. After assigning COS markers that are polymorphic between the chromosomes of wheat and the perennial grasses they will also be suitable for identifying the chromatin of grass species introgressed into wheat, and for marker-assisted selection to facilitate the transfer of useful agronomic traits.

## Conclusions

The comprehensive analysis of the genetic relationships between diploid wheatgrass species at the chromosomal- and DNA sequence level will facilitate the understanding of their evolutionary role within the *Triticeae* tribe. COS marker-based phylogenetic studies confirmed close genetic relationship between J and E genomes, while FISH using repetitive DNA probes proved to be suitable for characterization of individual J-, P-, and St genome chromosomes. Based on these detailed FISH-based molecular cytogenetic analysis and the selection of representative, polymorphic COS-markers of three diploid species will support the pre-breeding programmes of wheat aimed to utilize the genetic diversity of perennial wheatgrasses.

## Supporting information

S1 Table PCR products of COS markers. PCR products of COS markers amplified from total genomic DNA (gDNA) of wheat (GK Öthalom), *Thinopyrum elongatum* (MvGB 1963), *Thinopyrum bessarabicum* (MvGB 1706), *Pseudoroegneria spicata* (MvGB 1617), *Pseudoroegneria spicata* (MvGB 1607), *Pseudoroegneria spicata* (MvGB 1615), *Agropyron cristatum* (MvGB 1521) and *Agropyron cristatum* (MvGB 1509).
(XLSX)
